# Efficacy of retroperitoneal laparoscopic ureterolithotomy for the treatment of large proximal ureteric stones and its impact on renal function

**DOI:** 10.1186/2193-1801-2-600

**Published:** 2013-11-11

**Authors:** Takahiro Yasui, Atsushi Okada, Shuzo Hamamoto, Kazumi Taguchi, Ryosuke Ando, Kentaro Mizuno, Yasunori Itoh, Keiichi Tozawa, Yutaro Hayashi, Kenjiro Kohri

**Affiliations:** Department of Nephro-urology, Nagoya City University Graduate School of Medical Sciences, 1 Kawasumi, Mizuho-cho, Mizuho-ku, Nagoya, 467-8601 Japan

**Keywords:** Ureterolithotomy, Laparoscopic surgery, Retroperitoneal approach, Endourology, Renal function, 99mTc-MAG3

## Abstract

**Purpose:**

The purpose of this study was to evaluate the efficacy of retroperitoneal laparoscopic ureterolithotomy for the management of large proximal ureteric stones and the impact of this treatment on postoperative renal function.

**Methods:**

The data of 12 patients (7 men and 5 women; mean age, 68.5 ± 8.9 years) with large pyeloureteral junction (2 cases) and upper ureteral (10 cases) stones (25.3 ± 7.4 mm) that had undergone retroperitoneal laparoscopic ureterolithotomy were reviewed. Renal function was analyzed by the estimated glomerular filtration rate (eGFR) and renal scintigraphy using 99mTc-mercaptoacetyltriglycine (99mTc-MAG3) before and 3 months after surgery.

**Results:**

The mean operative time was 129.5 ± 21.4 minutes, with a mean blood loss of 64.4 ± 78.2 mL. The mean duration of hospital stay after surgery was 6.4 ± 2.7 days, and the mean duration of stenting was 7.2 ± 1.7 weeks. A stone clearance rate of 100% was achieved, and no patient developed ureteric stricture. 99mTc-MAG3 scintigraphy showed that laparoscopic removal of calculi did not affect renal function, but did improve ureteral occlusion.

**Conclusions:**

Retroperitoneal laparoscopic ureterolithotomy is a safe and effective treatment option for reducing ureteral obstruction in select patients with large proximal ureteric stones.

## Introduction

The advent of minimally invasive therapies in the form of endoscopic surgery and shock wave lithotripsy has revolutionized the treatment of urinary lithiasis over the last three decades and diminished the role of open stone surgery (Muslumanoglu et al. 2006). Laparoscopic ureterolithotomy is now an established alternative to open ureterolithotomy for the primary treatment of large, impacted, proximal or mid-ureteral stones or as a salvage procedure for failed cases of extracorporeal shock wave lithotripsy and attempted ureterorenoscopy of stones in these locations (Anagnostou & Tolley 2004; Wolf 2007). To our knowledge, although calculi with urinary obstruction lead to renal dysfunction and should be removed, the impact of laparoscopic lithotomy on renal function has not been considered in detail. Here, we evaluated the efficacy and safety of retroperitoneal laparoscopic ureterolithotomy for the management of large proximal ureteric stones and the impact of this treatment on postoperative renal function, using 99mTc-mercaptoacetyltriglycine (99mTc-MAG3), in patients who had undergone retroperitoneal laparoscopic ureterolithotomy.

## Patients and methods

### Ethics statement

All subjects provided written informed consent. The study protocol conformed to the Declaration of Helsinki and was approved by the ethical committees at the Nagoya City University Graduate School of Medical Sciences.

### Patients

The data of 12 patients (7 men and 5 women), with pyeloureteral junction (2 cases) and upper ureteral (10 cases) stones, who had undergone retroperitoneal laparoscopic ureterolithotomy between September 2010 and January 2012 at Nagoya City University were reviewed. The mean age of the patients was 68.5 ± 8.9 (range, 52–81 years). The stones were on the right side in 6 cases (50%) and on the left side in 6 cases (50%). The inclusion criteria were as follows: single large or impacted stone that indicated open surgery in 10 cases (83%) and unsuccessful previous shock wave lithotripsy (SWL) trial (no signs of disintegration after 2 sessions) in 2 cases (17%). Two cases of unsuccessful previous shock wave lithotripsy trial had not so enlarged stones (16 mm and 19 mm diameter).

### Operative technique

**Figure 1:**
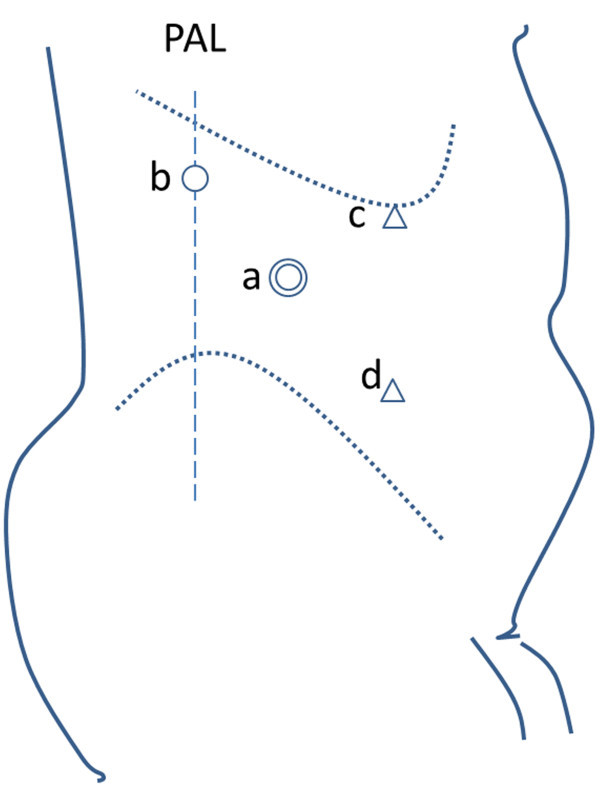
**Position of trocars during surgery. (a)** A 10-mm camera trocar above the anterior superior iliac spine on the mid-axillary line; **(b)** A 10-mm trocar below the 12^th^ rib on the posterior axillary line; **(c)** A 5-mm trocar the 12^th^ rib on the anterior axillary line; **(d)** A 5-mm trocar on the anterior axillary line above the iliac crest. PAL: posterior axillary line.

The patient was administered general anesthesia, and cystoscopy and ureteral catheterization were performed. The ureteral catheter was placed just distal to the stone in all cases, except in those wherein a double pigtail stent was previously inserted. After placement of the double pigtail stent, the patient was positioned in the flank position. The procedure was performed using the balloon technique of retroperitoneoscopy with the patient in the lateral kidney position. A 12-mm surface incision was made just distal and anterior to the 12^th^ rib on the midclavicular line. The muscles were split to expose and incise the dorsolumbar fascia. The peritoneum was pushed anteriorly by blunt finger dissection to create retroperitoneal space. The space was further enlarged using the indigenous balloon dissection technique. After the creation of a pneumoretroperitoneum, a 10-mm port was established for the camera, and CO_2_ pressure was maintained at 12 mm Hg. A 10-mm working port was established below the 12^th^ rib on the posterior axillary line, and a 5-mm working port was established below the 12^th^ rib on the anterior axillary line using laparoscopic vision. The third 5-mm working port was established on the anterior axillary line above the iliac crest (Figure 1).

**Figure 2:**
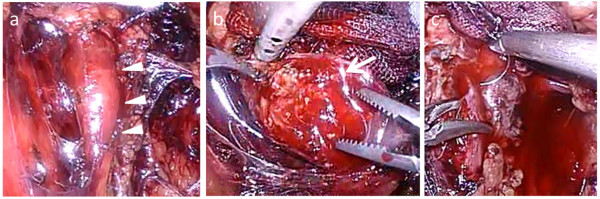
**Retroperitoneal laparoscopic ureterolithotomy. (a)** Stone bulge (arrowheads); **(b)** Stone removal (arrow); **(c)** Intracorporeal ureterotomy suturing.

The dissection was initiated by identification of the ureter with blunt dissection and recognition of the stone bulge (Figure 2A). A ureterotomy was then performed over the stone, and the stone was extracted (Figure 2B). The ureterotomy incision was closed using 4–0 PDS (Ethicon Endo-Surgery, Cincinnati, OH, USA) as an interrupted suture (Figure 2C). A 15-Fr soft silastic drain was then inserted, and port site closure was completed.

### Postoperative course and care

Patients were administered intravenous fluid until the presence of bowel sounds was noted. Abdominal radiography was performed to confirm the position of the double pigtail stent and residual stones. On postoperative day 2, renal ultrasonography was used to assess the underlying hematoma or collections, and the drainage tube was removed if the patient’s 24-hour output was less than 50 mL. The double pigtail stent was removed after 6–8 weeks, and the patient was administered antibiotic therapy. Excretory urography was performed at 3 months after surgery. Subsequent follow-up was performed at 6 months and then annually. At each visit, serum creatinine levels were determined, and renal and bladder ultrasonographies were performed. Excretory urography was indicated if increasing hydroureteronephrosis was noted or if patients had symptoms of pain.

### Renal function evaluation

Renal function was analyzed using the estimated glomerular filtration rate ([eGFR] = 194 × serum creatinine^-1.094^ × age^-0.287^ × [1 - gender × 0.261], where gender was 0 for male and 1 for female) and renal scintigraphy with 99mTc-MAG3 before and 3 months after surgery. Affected renal function was evaluated by the ratio of affected renal 99mTc-MAG3 clearance to contralateral renal 99mTc-MAG3 clearance. Renal obstruction was determined by evaluating the perfusion time-activity curves and the time from peak to 50% activity (T1/2) by using a renogram.

### Statistical analyses

Statistical analyses were performed using SPSS software version 15.0 (IBM, Armonk, NY, USA). The Fisher exact test, Mann–Whitney *U* test, and the Pearson correlation were used to assess different continuous variables, with p values < 0.05 considered statistically significant.

## Results

Procedures were completed laparoscopically without the need for an open operation. The total mean operative time, calculated from trocar placement to skin closure without double pigtail stent placement, was 129.5 ± 21.4 minutes (range, 96–170 minutes). The mean blood loss (including urine) was 64.4 ± 78.2 mL (range, 3–212 mL), and the mean stone size (diameter) was 25.3 ± 7.4 mm (range, 16–40 mm). With regard to the composition of stones, 10 cases had calcium oxalate stones, whereas 2 cases had struvite stones. The drain was removed 2–3 days after surgery, although a double pigtail stent had to be re-inserted 3 days post-operation in one patient with persistent urine discharge because of stent obstruction. Leakage then stopped without any consequences. The average hospital stay after surgery was 6.4 ± 2.7 days, with a mean time to normal activity of 1.7 weeks (range, 1–3 weeks) and a mean stenting duration of 7.2 ± 1.7 weeks. At follow-up, all patients were symptom-free. Postoperative excretory urography did not reveal any evidence of obstruction in any patients.

**Figure 3:**
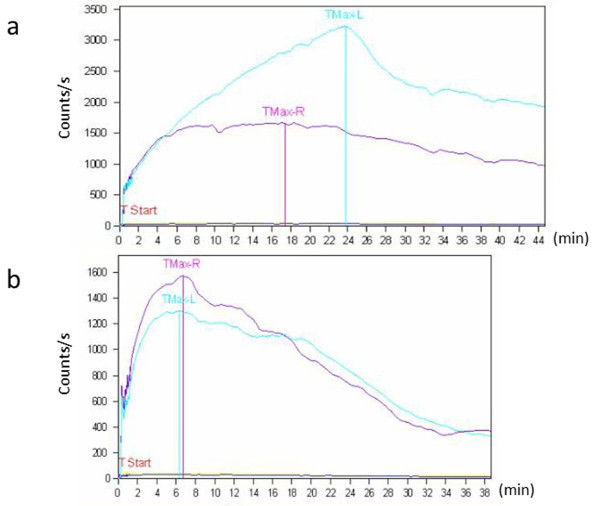
**Renogram of renal scintigraphy with 99mTc-mercaptoacetyltriglycine. (a)** before and **(b)** 3 months after surgery. The time from peak to 50% activity (T1/2) of this case improved from immeasurable to measurable. Tmax, time until peak activity.

Renal function was assessed preoperatively and postoperatively by using serum creatinine levels, eGFR, and renal 99mTc-MAG3 scintigraphy (Table 1). Preoperative and postoperative total renal function, estimated by serum creatinine levels (0.87 ± 0.31 and 0.78 ± 0.30 mg/dl, respectively) and eGFR (66.0 ± 18.39 and 75.2 ± 23.2 ml · min^-1^ · 1.73 m^-2^, respectively), showed no significant change In addition, preoperative and postoperative affected renal function, evaluated by affected/contralateral renal 99mTc-MAG3 clearance in 10 patients (0.77 ± 0.19 and 0.80 ± 0.18, respectively), also showed no significant change (101.8% ± 5.5% postoperatively compared to 100% preoperatively). In contrast, postoperative affected ureteral obstruction, evaluated based on the time from peak activity to T1/2, did show changes. In 2 patients, the T1/2 value improved from 18.8 to 14.8 minutes and from 10.2 to 6.3 minutes, whereas in 4 patients, the T1/2 value improved from immeasurable to measurable (Figure 3). Additionally, 4 patients showed a tendency toward improvement based exclusively on the 99mTc-MAG3 scintigraphy curve. These results indicate that laparoscopic removal of calculi did not affect renal function, but did improve ureteral occlusion.

**Table 1:** **Preoperative and postoperative renal function in a case undergoing retroperitoneal laparoscopic ureterolithotomy**

	Preoperative value	Postoperative value	
Serum creatinine (mg/dl)	0.87 ± 0.31	0.78 ± 0.30	N.S.
eGFR (ml · min^-1^ · 1.73 m^-2^)	66.0 ± 18.39	75.2 ± 23.2	N.S.
Affected/contralateral renal 99mTc-MAG3 clearance (compared to the preoperative value, %)	-	101.8 ± 5.5	N.S.

99mTc-MAG3, 99mTc-mercaptoacetyltriglycine, eGFR, estimated glomerular filtration rate; N.S., no significant difference.

## Discussion

Laparoscopic urological surgery is increasingly replacing open surgery as a result of accumulated surgical experience. Laparoscopy is associated with lower postoperative morbidity, shorter hospital stays and time to convalescence, and better cosmetic results with comparably good functional results (Skolarikos et al. 2010; Hruza et al. 2009; Hemal et al. 2003). In the present era of minimally invasive surgery, laparoscopic ureterolithotomy remains a valuable alternative to open lithotomy for the primary treatment of impacted upper and mid-ureteral stones larger than 1.5 cm (Gaur et al. 2002; Leonardo et al. 2011). Laparoscopic ureterolithotomy is relatively easy, with good results. The distinct advantage of laparoscopic ureterolithotomy is the high probability of removing the entire stone in one procedure, which is economical and ensures shorter operation times with indwelling ureteral stents compared to extracorporeal SWL performed using a stent in the renal unit (Goel & Hemal 2001; Skrepetis et al. 2001). However, laparoscopic ureterolithotomy is not suitable when stone had been crushed by extracorporeal SWL, because complete removal for shattered stones is difficult. Two cases after SWL in this study have been affected no effect by previous SWL.

Laparoscopic ureterolithotomy can be performed via the transperitoneal or retroperitoneal route (Skolarikos et al. 2010; Leonardo et al. 2011; Wang et al. 2010; Singh et al. 2013; Farooq Qadri et al. 2011). Simforoosh et al. compared the use of a retroperitoneal versus intraperitoneal approach for laparoscopic proximal ureterolithotomy and reported that operative time was significantly different in favor of the intraperitoneal approach (Simforoosh et al. 2007). Moreover, Sing et al. compared the approach routes in laparoscopic ureterolithotomy and concluded that treatment of proximal, mid-ureteral, large, and impacted stones with transperitoneal laparoscopic ureterolithotomy is associated with greater pain, greater tramadol requirement, ileus, and longer hospital stays compared to retroperitoneal laparoscopic ureterolithotomy (Singh et al. 2013). Based on these findings, the retroperitoneal approach should be the procedure of choice for these types of stones.

Here, we performed laparoscopic ureterolithotomy via the retroperitoneal route. Proper patient selection allowed for completion of the procedure without the need for open conversion and indicates that a favorable surgical outcome depends on a combination of proper patient selection and surgical experience. In line with this observation, the mean operative time was approximately 130 minutes within a range of 96–170 minutes. However, one initial case required a longer operative time up to 150 minutes, and 2 cases with severe adhesion around the ureter after pyelonephritis required a longer time of 140–170 minutes. Thus, the actual range was 90–130 minutes, with a mean time of approximately 90 minutes.

A major complication of laparoscopic ureterolithotomy is ureteral stricture, which has been reported in 15–20% of cases in a separate series. Although the etiologies of postoperative ureteral strictures are not clear, explanations involve tight suturing of the ureterotomy leading to wall ischemia with subsequence stenosis. In addition, prolonged postoperative urinary drainage with retroperitoneal fibrosis is another possible cause of ureteral stenosis (Mitchinson & Bird 1971). However, Nouria et al. estimated the incidence of ureteral stricture to be only 2.5% based on a review of previously published reports (Nouira et al. 2004), and none of our cases experienced ureteral stricture after surgery.

In the present study, all patients were discharged stone-free without stone recurrence throughout the follow-up period and were free of complications, indicating the safety and efficacy of a laparoscopic retroperitoneal approach. In fact, the stone-free rate for laparoscopic lithotripsy is reportedly higher than that for ureterorenoscopic or percutaneous nephrolithotripsy (Basiri et al. 2008; El-Moula et al. 2008). Laparoscopic ureterolithotomy is relatively easy, with stone-free rates up to 100%, provided that expertise is available (Fan et al. 2009; Khaladkar et al. 2009). However, laparoscopic ureterolithotomy in the distal ureter is less successful than in the middle and proximal ureter, although the size of the stone does not appear to influence outcome. According to the European guidelines, laparoscopic ureterolithotomy is not first-line therapy in most cases, the indication of laparoscopic ureterolithotomy is limited for large or impacted stone.

Recoverability of renal function after obstruction depends on the level, the duration of the obstruction. Importantly, removal of stones did not cause either deterioration or improvement of the affected-side kidney function; however, improvement of obstruction was confirmed by 99mTc-MAG3 scintigraphy, indicating that removal of large stones is useful for maintaining renal function in the future.

## Conclusion

Retroperitoneal laparoscopic ureterolithotomy is a safe and effective treatment option for select patients with large and impacted proximal ureteric stones and can be used as a salvage procedure for SWL or ureteroscopy. The procedure has all of the advantages of laparoscopy, including good cosmetic appearance and a short convalescence period. However, considering the limitations of the retrospective and non-competitive design of our study, larger scale prospective randomized controlled trials are mandatory to confirm the therapeutic yield of this option.
